# Transcatheter Closure of Perimembranous Ventricular Septal Defect with Aneurysm: Radiologic Characteristic and Interventional Strategy

**DOI:** 10.1155/2020/6646482

**Published:** 2020-12-24

**Authors:** Weibing Guo, Yifan Li, Jinjin Yu, Junjie Li, Ling Sun, Jijun Shi, Shushui Wang, Hong Chen, Zhiwei Zhang

**Affiliations:** ^1^Guangdong Cardiovascular Institute, Guangdong Provincial People's Hospital, Guangdong Academy of Medical Sciences, Guangdong, China; ^2^Department of Pediatric Cardiology, Guangdong Provincial People's Hospital, Guangdong Academy of Medical Sciences, Guangdong Cardiovascular Institute, Guangdong, China; ^3^Shantou University Medical College, Guangdong Provincial People's Hospital, Guangdong Academy of Medical Sciences, Guangdong, China

## Abstract

**Objectives:**

We aimed to explore the radiologic characteristics and interventional strategies for perimembranous ventricular septal defect (pmVSD) with aneurysm.

**Methods:**

257 patients who underwent transcatheter closure of pmVSD with aneurysm were included in our study. We retrospectively reviewed the left ventricular opening of the aneurysm (a), diameter of the midsegment of the aneurysm (b), and diameter of the right ventricular opening of the aneurysm (c). If there were multiple defects within the aneurysm, the largest defect was denoted as *c*_1_ and so forth. We developed a novel VSD classification method in which pmVSD with aneurysm was classified into three types (A, B, and C). When *a* >*b* ≥ *c*, it was classified as type A, when *b* > *a* ≥ *c*, it was type B, and when *c* > *a* ≥ *b*, it was type C; *c*/*c*_1_ described the relationship among defects.

**Results:**

All of the 257 cases of pmVSD with aneurysm were defined using left ventriculography: type A, 60, type B, 58, and type C, 139. Transcatheter closure was attempted in 244 patients and succeeded in 227 cases (success rate was 93.0%; 227/244). Forty symmetric VSD occluders and 13 asymmetric VSD occluders were used for type A aneurysm occlusion; 31 symmetric VSD occluders, 19 asymmetric VSD occluders, and one Amplatzer duct occluder II (ADOII) were used for type B; 59 VSD symmetric occluders, 59 asymmetric VSD occluders, three eccentric VSD occluders, and two ADOII were used for type C. Within 24 hours after procedure, 2.2% patients had postprocedural residual shunt, and 2.2% experienced malignant arrhythmia (including type II second-degree AVB, cAVB, and CLBBB). Two hundred and twelve patients completed follow-up (93%, 212/227). No new severe complications were reported during follow-up, except in one patient who underwent surgery (removal of the device, VSD repair, and tricuspid valvuloplasty) due to severe postprocedural tricuspid regurgitation.

**Conclusions:**

It is safe and effective to apply this method for the classification of pmVSD with aneurysm and its interventional strategy.

## 1. Introduction

Perimembranous ventricular septal defect (pmVSD) is the most common type of ventricular septal defect (VSD). Aneurysm of the ventricular septum is thought to be related to spontaneous closure of a VSD, and more than 30–60% of pmVSD are associated with aneurysm [1–3]. Previous studies have shown that most aneurysms associated with VSD are pseudoaneurysms, which are constituted by the tricuspid valve and surrounding proliferating fibrous tissue [4]. The complex relationship with the tricuspid valve and surrounding tissue, various morphologies, and uneven sizes of the defects pose great challenges to interventional therapy for pmVSDs with aneurysm. Numerous studies have focused on occlusion strategies of this specific type of VSD; however, most of them have only discussed the Amplatzer duct occluder I (ADOI, Abbott, USA) and the Amplatzer duct occluder II (ADOII, Abbott, USA) [5–8]. Nevertheless, these two types of occluders cannot meet the needs of clinical practice. The rapid development of domestic occluders in China has provided more choices for the closure of this type of VSD [9–11]. This study aimed to explore the radiologic characteristics and interventional strategies for pmVSD with aneurysm.

## 2. Materials and Methods

### 2.1. General Information

From November 2016 to June 2018, 257 patients were diagnosed with pmVSD with aneurysm and were included in our study.

The inclusion criteria were as follows: (1) indications for intervention guidelines [12], including age ≥2 years or weight ≥10 kg, and no association with severe aortic prolapse reflux or regurgitation; (2) diagnosis with pmVSD with aneurysm by intraoperative left ventricular (LV) angiography (*h* ≥ 3 mm).

The exclusion criteria were as follows: (1) moderate to severe pulmonary hypertension; (2) other congenital heart defects; (3) other systemic diseases requiring medical treatment.

This study was approved by the ethics committee and was exempted from the need for informed consent.

### 2.2. Method of Classification

Different types of pmVSD with aneurysm were identified according to their morphology, as shown by left ventriculography in a single plane (60° left anterior oblique/20° cranial). The height of the aneurysm (*h*), LV opening of the aneurysm (a) (i.e., the inlet portion of the aneurysm), diameter of the midsegment of the aneurysm (b), and diameter of the right ventricular (RV) opening of the aneurysm (c) (i.e., the outlet portion of the aneurysm) were measured. If there were more than one defect within the aneurysm, the largest defect was denoted as *c*_1_ and so forth. Three types of aneurysms were classified according to *a*, *b*, and *c*.

**Figure 1:**
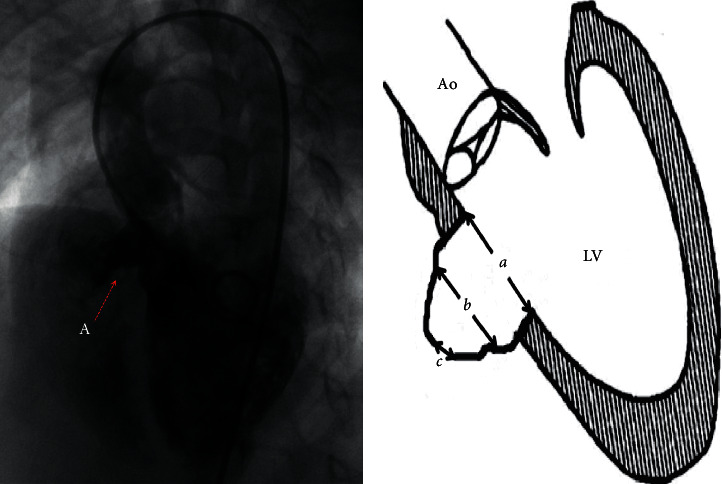
Angiography of type A aneurysm and schematic diagram of type A aneurysm. *a* is the inlet portion of the aneurysm, *b* is the diameter of the midsegment of the aneurysm, and *c* is the outlet portion of the aneurysm.

**Figure 2:**
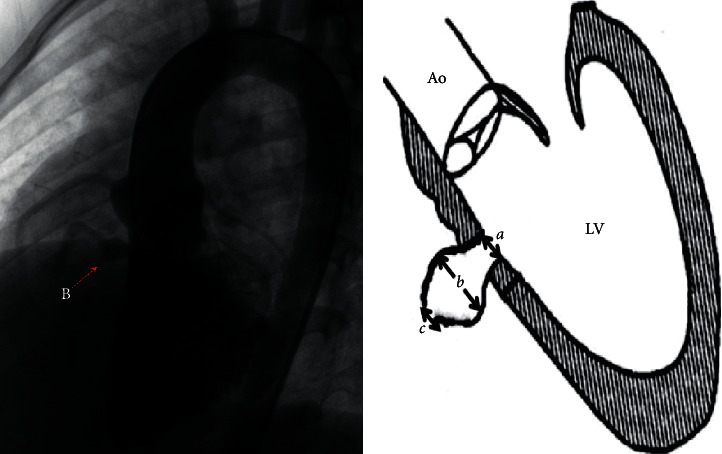
Angiography of type B aneurysm and schematic diagram of type B aneurysm. *a* is the inlet portion of the aneurysm, *b* is the diameter of the midsegment of the aneurysm, and *c* is the outlet portion of the aneurysm.

**Figure 3:**
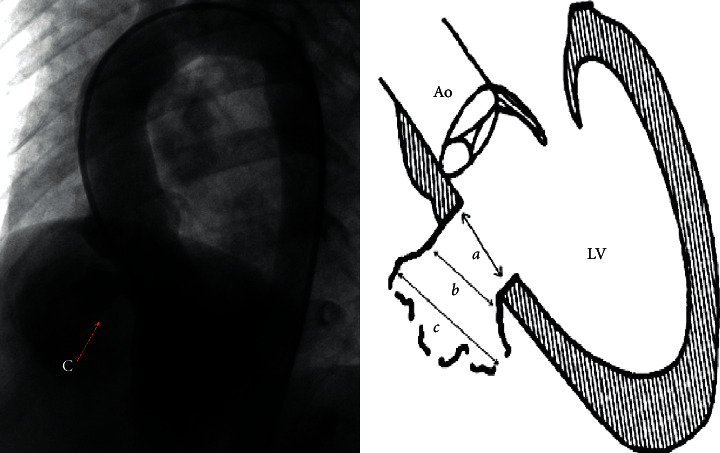
Angiography of type C aneurysm and schematic diagram of type C aneurysm. *a* is the inlet portion of the aneurysm, *b* is the diameter of the midsegment of the aneurysm, and *c* is the outlet portion of the aneurysm.

When *a* >*b* ≥ *c*, it was classified as type A. This type of aneurysm has a relatively large inlet portion of the aneurysm, and as the aneurysm extended, the outlet portion of the aneurysm tapers. The RV opening of the aneurysm is restrictive, usually with single or multiple defects, and if multiple defects exist, they are close to each other (Figure 1). When *b* > *a* ≥ *c*, it was classified as type B, which has relatively small inlet or outlet portions, but the aneurysm bulges in to the RV like a pouch (Figure 2). When *c* > *a* ≥ *b*, it was classified as type C. Type C is larger than the other types and usually has fenestrated shunts on the right side. If there is a large inlet portion and a small subaortic rim, the closure device must be retrieved due to significant residual shunt, and the patient must receive surgical repair (Figure 3).

*c*/*c*_1_ was used to describe the relationship among defects; a larger *c*/*c*_1_ indicates smaller and more defects within the aneurysm.

### 2.3. Occluder Device

**Figure 4:**
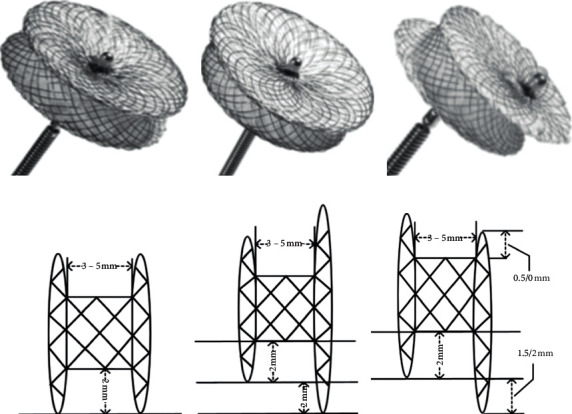
Occluders and structure diagrams. The symmetric occluder (a, d); the asymmetric occluder (b, e); the eccentric occluder (c, f).

Four types of closure devices were used, including ADOII (Abbott, USA) and three types of double-disk occluder (Lifetech Scientific Shenzhen Co. Ltd, Shenzhen, China; Starway Medical Technology, Inc., Beijing, China): symmetric occluder(A2B2), asymmetric occluder (A4B2), and eccentric occluder. All three types of occluder have the same right disk, which is 4 mm larger than the waist. Besides, the left disk of the symmetric occluder is 4 mm larger than the waist, the left disk of the asymmetric occluder is 8 mm larger than the waist, and the eccentric occluder has a special eccentric left disk; that is, the side towards to the aortic valve is 0 mm or only 0.5 mm, and another side is 4 mm or 3.5 mm (Figure 4).

### 2.4. Procedure and Perioperative Management

**Figure 5:**
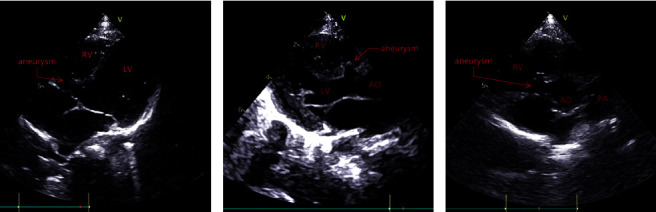
The VSD with aneurysm in the apical five-chamber view (a), the long-axis parasternal (b), and the short-axis parasternal view (c) of TTE.

All patients underwent transthoracic echocardiography (TTE) and electrocardiography (ECG) before the procedure. The VSD with aneurysm was evaluated at the apical five-chamber view, short-axis parasternal view, and long-axis parasternal view (Figure 5). All patients received prophylactic antibiotics (cefazolin sodium pentahydrate or clindamycin) once before the procedure.

The procedure was performed under general anesthesia in pediatric patients. Puncture of both the femoral artery and vein was performed for vascular access. Heparin (100 IU/kg) was injected intravenously during the procedure. A complete hemodynamic evaluation was performed, including the pulmonary flow (*Qp*)/systemic flow (*Qs*) ratio and pulmonary vascular resistance. The location, shape, and size of the VSD was confirmed by left ventriculography and TTE during the procedure.

**Figure 6:**
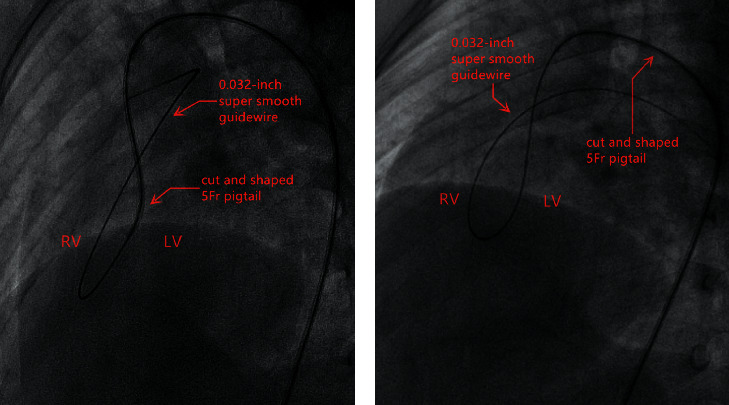
Establishing the orbit by inserting the catheter into the VSD (a) or operating the guidewire to traverse the VSD directly (b).

A partially cut and shaped 5Fr pigtail and a 0.032 inch super smooth guidewire were used to establish an arteriovenous loop (A-V loop). Two methods used to establish the A-V loop: (1) the guidewire was drifted with the direction of flow through the VSD to the RV from the LV directly; (2) the catheter was inserted into the VSD from the LV; the guidewire was pushed out of the outlet to the RV through the catheter (Figure 6). The long sheath was advanced through the A-V loop into the LV from the femoral vein. The occluder was loaded into the long sheath and was sent to the LV. After the left disk and waist of the occluder was opened, the entire system was withdrawn to the RV and the right disk was released. Before the occluder was finally released, left ventriculography and TTE was performed again to evaluate the position and shape of the occluder, residual shunt, significant regurgitation (AR) aortic valve, and tricuspid valve regurgitation (TR) (increased regurgitation area should be <2 cm^2^).

Intramuscular injection of low molecular weight heparin was administered at 6 hr and 12 hr after the procedure, and aspirin was prescribed 3–5 mg/kg daily for six months.

### 2.5. Selection of the Occluder

**Figure 7:**
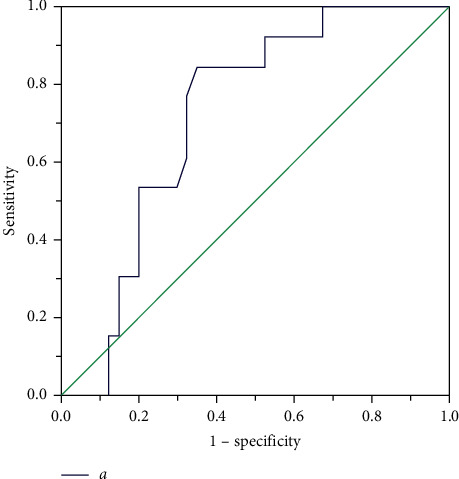
ROC curve describing the relationship between the inlet portion of the aneurysm and occluder in type A aneurysm.

Type A: when inlet (a) <8.54 mm, a symmetric occluder was usually selected for occlusion; the left disc of the occluder was implanted at the inlet, and the waist and right disk were in the aneurysm. The waist of occluder was selected as 2–4 mm larger than (a). When (a) ≥8.54 mm, we tended to select an asymmetric occluder (Figure 7); the left disc was implanted into the aneurysm, the waist was at the outlet, and the right disk was in RV. The size of the occluder was chosen based on the diameter of the midsegment of the aneurysm (b). The left disc of the occluder should be 3-4 mm larger than (b).

**Figure 8:**
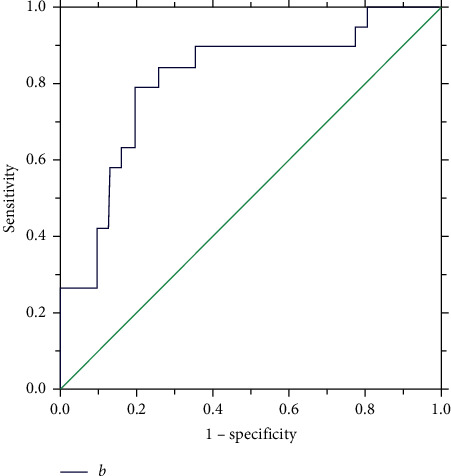
ROC curve describing the relationship between the diameter of the midsegment of the aneurysm and occluder in type B aneurysm.

Type B: when (a) <6.1 mm, we selected the symmetric occluder and implanted it at the inlet. The size of the selected occluder was the same as (a). When (a) ≥6.1 mm, the left disk was implanted into the aneurysm; the left disk of the occluder should be 3-4 mm larger than (b). However, the selection of occluder type was significantly affected by (b) (*t* = 2.494; *p* < 0.05) in the numerous factors (Figure 8). When (b) ≥9.04 mm, an asymmetric occluder was applied.

**Figure 9:**
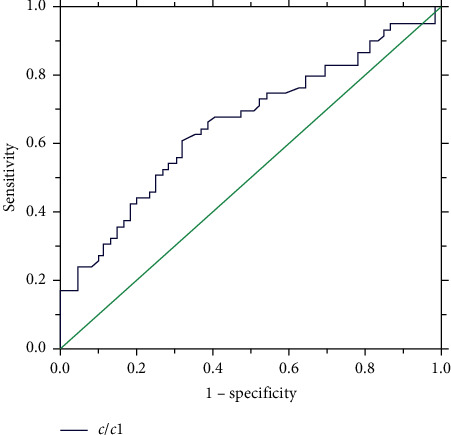
ROC curve describing the relationship between the value of *c*/*c*1 and occluder in type C aneurysm.

Type C: when (a) <6.1 mm, we selected the symmetric occluder and implanted it at the inlet. The size of the selected occluder was the same as (a). When (a) ≥6.1 mm, the *c*/*c*_1_ was the decisive parameter (*t* *=* 2.367, *p* < 0.05) in choosing the occluder type. When *c*/*c*_1_ ≥ 3.69 (Figure 9), we were inclined to use an asymmetric occluder. Otherwise, a symmetric occluder is better. In this condition, the left disk of the occluder should be 3-4 mm larger than (c).

**Figure 10:**
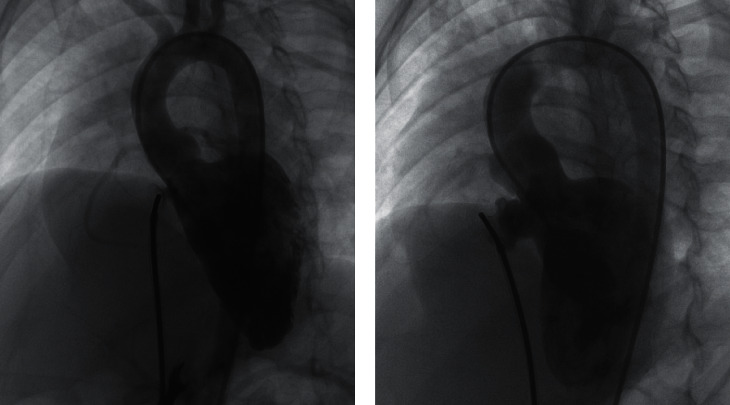
Left ventriculography with occluder implanted at inlet (a) and occluder implanted into aneurysm (b).

As for those who had an angiography showing an inlet portion of the aneurysm close to the aortic valve, an eccentric occluder or Amplatzer Duct Occluder II (ADOII) was considered the best choice (Figure 10).

### 2.6. Follow-Up

All patients with successful closure underwent TTE and ECG within 24 hr after the procedure. All patients were followed up at 1, 3, 6 and 12 months after the procedure and on a yearly visit thereafter. We focused on the presence of residual shunt and arrhythmia. For those who were found to have arrhythmia within the 24 hr, ECG was performed again before discharge.

Malignant arrhythmia, including type II second-degree atrioventricular block (AVB), complete atrioventricular block (cAVB), and complete left bundle branch block (CLBBB), a significant increase in valvular regurgitation (≥2 cm^2^), and residual shunt ≥2 mm with cardiac murmur were defined as major adverse events.

### 2.7. Statistics

All data were processed with SPSS 22.0 software (IBM Corporation; Somers, NY). Means and standard deviations (SD) were reported for all measurement data, and the variance test was used to analyze significance. The degree of influence of various factors was analyzed using ridge regression analysis. The critical value was obtained by fitting the ROC curve. *p* < 0.05 was considered statistically significant.

## 3. Results

A total of 257 patients were included in our study, accounting for 36.5% of all types of VSDs in the same period. 134 patients were male (52.3%), and 123 were female (47.7%). The mean ages and weights were 4.18 ± 3.68 years and 16.06 ± 6.86 kg, respectively. The average *Q*p/*Q*s ratio was 1.59 ± 0.45, and the mean pulmonary artery pressure was 29.4 ± 7.6/12.4 ± 5.3 mmHg. A total of 188 (73.2%) were diagnosed preoperatively with pmVSD with aneurysm, and preprocedural echocardiography showed that the average diameter of the LV opening of the aneurysm was 6.59 ± 2.56 mm (0.60 – 15.40 mm). There was no statistical difference between the diameter of the LV opening measured by TTE and that measured by angiography (*p*=0.318).

### 3.1. Type of pmVSD Aneurysm

60 cases were classified as type A, 58 cases, as type B, and 139 cases, as type C. There was a significant statistical difference among the radiologic data of the three types of aneurysm (Table 1).

### 3.2. Occlusion Results

The occlusion was attempted in 244 patients (244/257). Thirteen patients (13/257) were considered not suitable for transcatheter closure due to following reasons: oversize defect (six cases), preoperative underestimated aortic valve prolapse (four cases), and trivial shunt requiring no intervention (three cases).

The devices were successfully deployed in 227 cases, while 17 patients who failed to have a successful device closure underwent surgical repair. Therefore, the immediate procedure success rate was 93.0% (227/244). Of those who had successfully occlusion, the average procedure time was 37.13 ± 17.04 min (16.33 – 121.58 min). Six patients (2.6%) underwent retrieval of the occluder or adjustment of device position because of unsatisfying device deployment in the first attempt. The causes of failure were significant residual shunt (eight cases), severe aortic regurgitation (two cases), and severe malignant arrhythmia (seven cases) during the procedure.

53 successful cases were type A (88.3%, 53/60). A symmetric occluder was deployed in 40 cases (75.4%, 40/53), and an asymmetric occluder was deployed in 13 cases (24.6%, 13/53). In 32 cases (60.4%, 32/53), the devices were anchored at the LV opening of the aneurysm (i.e., the inlet portion of the aneurysm). In the remaining 21 cases (39.6%, 21/53), the device was anchored at the RV opening of the aneurysm (i.e., the outlet portion of the aneurysm). The left disk of the occluder was pulled into the aneurysm, and the device was situated in the aneurysm.

As for type B, there were 51 successful cases (87.9%, 51/58). A symmetric occluder was deployed in 31 cases (60.8%, 31/51), an asymmetric occluder was deployed in 19 cases (37.3%, 19/51), and ADOII was deployed in one case (2%, 1/51). In 31 cases (60.8%, 31/51), the devices were anchored at the inlet portion. In 20 cases (39.2%, 20/51), it was anchored at the outlet portion.

**Figure 11:**
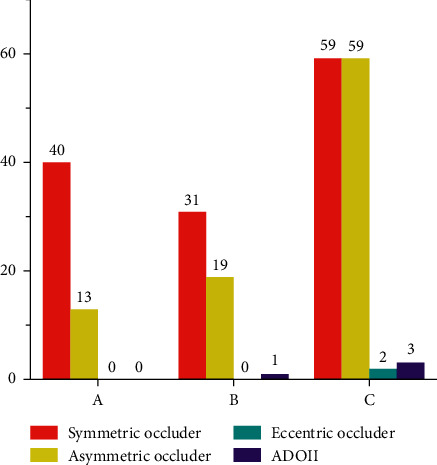
Application of different types of occluders in different types of aneurysm.

**Figure 12:**
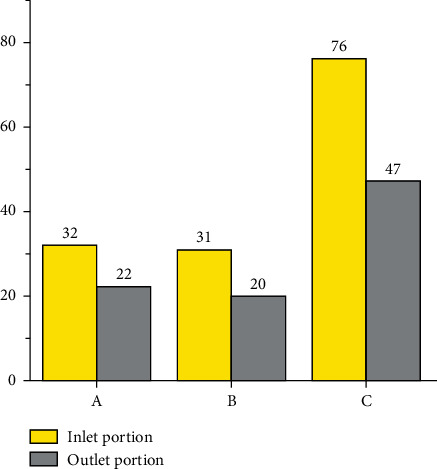
Implanted position of occluders in different types of aneurysm.

For type C, there were a total of 123 successful cases (88.5%, 123/139). A symmetric occluder was deployed in 59 cases (48%, 59/123), an asymmetric occluder was deployed in 59 cases (48%, 59/123), an ADOII was deployed in three cases (2.4%, 3/123), and an eccentric occluder was deployed in two cases (1.6%, 2/123). The devices were anchored at the inlet portion in 76 cases (61.8%), and 47 cases (38.2%) were anchored at the outlet portion (Figures 11 and 12).

### 3.3. Follow-Up

TTE and ECG were performed in these patients within 24 hr after the procedure. A residual shunt with murmur was detected in five cases (2.2%). TR was slightly increased in seven cases (3.1%), and trivial aortic regurgitation (AR) occurred in two cases (0.8%). Malignant arrhythmia occurred in five cases (2.2%), including one patient with type II second-degree AVB, one patient with cAVB, and three patients with CLBBB.

Two patients who experienced type II second-degree AVB and cAVB underwent removal of the device, and a temporary pacemaker was surgically implanted for supportive treatment. After one week of therapy with intravenous glucocorticoids (1 mg/kg, bid) and temporary cardiac pacing, these two patients recovered to complete right bundle branch block (CRBBB) and first-degree AVB, respectively. They were successfully discharged from the hospital. Three patients with CLBBB were discharged from the hospital without therapy and were advised that regular visits were necessary to evaluate the indications for further treatment.

A total of 212 patients completed follow-up (93%, 212/227), with an average follow-up time of 12.32 ± 8.23 months (range: 1–24 months). Fifteen patients were lost to follow-up. During follow-up period, a mild residual shunt was detected in 10 patients. Among them, five patients developed a new onset mild residual shunt at 1–3 months after the procedure. Arrhythmia persisted in five cases; however, cardiac function deterioration or chamber enlargement was not observed during follow-up. No delayed arrhythmia was detected.

**Figure 13:**
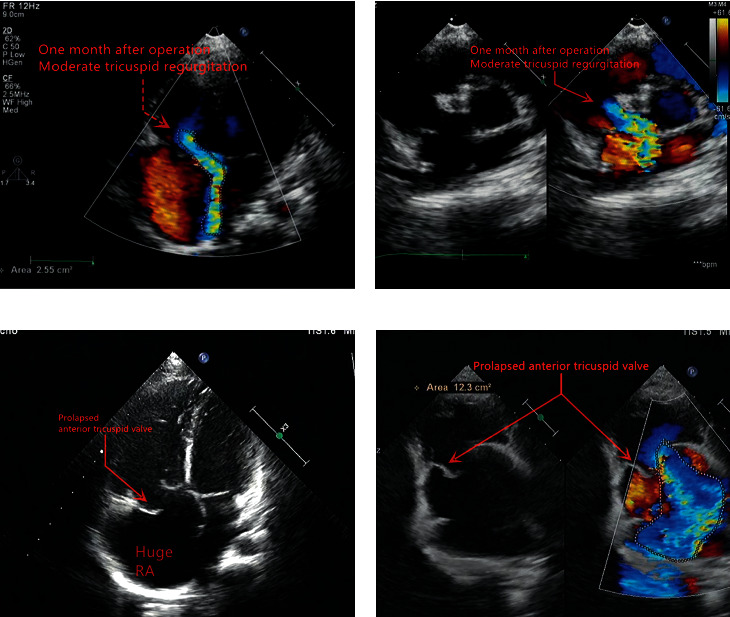
Deterioration in patients undergoing surgery for severe tricuspid regurgitation: one month after procedure (a-b); 36 months after procedure (c-d).

Valvular regurgitation observed immediately after the procedure did not deteriorate through the follow-up period. No new onset valvular regurgitation was observed during the follow-up, except for one patient who had to undergo surgery (removal of the device, ventricular septal defect repair, and tricuspid valvuloplasty) at 36 months after the procedure, due to severe TR (Figure 13). TR (2.6 cm^2^) was observed as early as one month after the procedure, which gradually increased to 10.5 cm^2^ at 36 months. At the same time, TTE also demonstrated right heart enlargement; therefore, surgery was unavoidable. During the surgery, rupture of the tricuspid valve papillary muscle was found, which was considered to be device-related.

No changes in heart function or anatomical structure were observed in other patients.

## 4. Discussion

PmVSD with aneurysm is a common form of VSDs, which means a balloon-like pouch or bulge was formed right at the defect, which can be divided into two categories: true aneurysm and pseudoaneurysm. According to the previous studies, pseudoaneurysm accounts for the majority of pmVSDs with aneurysm [13]. Various anatomical morphologies, complex relationships with the tricuspid valve and its surrounding tissues, and weak structure of the aneurysm make it easy to deform and even rupture during procedure, posing significant challenges to the occlusion of these defects. Most studies on pmVSD with aneurysm emphasized the risk factors of complications. Our team is the first team to use this novel quantitative method to classify pmVSD with aneurysm with the goal to aid in decision-making regarding interventions. Under guidance of this method, the incidence of malignant arrhythmia and residual shunt within 24 hr postoperatively was lower than that previously reported [14, 15]. No delayed arrhythmia was observed, and no further treatment was required for residual shunt during follow-up. Furthermore, no changes in heart function or chamber size were observed, except in one patient.

### 4.1. Residual Shunt

Residual shunt is the most common complication, particularly for pmVSD with aneurysm [16]. Residual shunt occurred immediately after the procedure in five patients. We speculated that this was related to position adjustment of the occluder. After the occluder was released, the tension from the cable was disappeared, and the implanted position of occluder may change slightly due to the excessive pressure from outlet tissues of the aneurysm. This pressure usually acts on the waist of the occluder, which eventually results in the phenomenon that the occluder could not be able to cover the defect completely. When a large left disk is required to completely close the aneurysm with a larger *c*/*c*_1_, the smaller waist of an asymmetric occluder receives less pressure than a symmetric occluder. Therefore, the application of an asymmetric occluder reduces the possibility of a change in the position of the occluder, thus reducing the risk of residual shunt. The lower incidence of residual shunt in this group supported our theory. In this study, we also found out that *c*/*c*_1_ was positively associated with (a) in type A (*r*_*s*_ = 0.35, *p* < 0.05), (b) in type B (*r*_*s*_ = 0.423, *p* < 0.01), and (c) in type C (*r*_*s*_ = 0.429, *p* < 0.05). These results further verified the rationality of our method.

Five patients had new onset residual shunt during follow-up. The most likely explanation was that the size of the aneurysm was enlarged due to the excessive pressure from the LV after closure of the outlet [17].

### 4.2. Arrhythmia

Previous researches have suggested that an oversized occluder can lead to a much higher risk of arrhythmia [18]. The friction and pressure between the left disk of the occluder and the bundle branch were deemed as potential reasons, and these factors always increase with the size of the occluder. When the inlet portion of the VSD is large, a larger ratio of the left disk to the inlet is required to achieve successful closure. By using our method, we chose to implant the occluder into the aneurysm when the inlet was large, which can avoid implantation of an oversized occlude, thereby reducing the risk of arrhythmia.

Meanwhile, after intraoperative angiography, the occluder can be selected more accurately using this method, which can avoid the damage to the bundle branch caused by repeated replacement of the occluder.

### 4.3. Valvular Regurgitation

Delayed severe tricuspid valve injury in one patient was surgically proven to be caused by the oversized right disk friction. This result indicated that the oversized right disk is an important factor causing device-related tricuspid valve injury. In this study, the application of an asymmetric occluder avoided the excessive right disk, thus reducing the possibility of tricuspid valve injury caused by this factor.

Actually, TR immediately after the procedure is considered to be more procedure-related than device-related. When the A-V loop was established, the excessive tension produced by the guidewire passing through the chordae tendineae or papillary muscles of the tricuspid valve caused injury or rupture of the chordae or papillary muscles, which was considered to be the main reason for TR. Increased heart rate (usually ≥10% of basic heart rate) is the most obvious manifestation when the tricuspid valve is affected during the procedure. It is important to observe and adjust the position of the guidewire in time to avoid tricuspid valve injury.

At the same time, the special structure of the eccentric occluder and soft material of ADOII were used in the VSDs that were close to the aortic valve in order to avoid aortic valve injury.

### 4.4. Success Rate

The success rate of this cases series was 93.0%. The main factors that affected the success rate of the procedure were the intraoperative malignant arrhythmia and residual shunt. One of the effective ways to improve the procedure success rate is to reduce the occurrence of severe arrhythmia during the procedure. According to our experience, when establishing the A-V loop, drifting the guidewire through the VSD to the RV from the LV may cause less arrhythmia than inserting the catheter into the VSD. If the application of a catheter is unavoidable, smoothing out the shaping area of catheter is also an important method. It is likely to be related to reducing catheter-induced stimulation to ventricular septum.

Rigorous screening procedure of patients by angiography is another effective way to improve the success rate. Of the failing cases with significant residual shunt, five patients (62.5%, 5/8) were diagnosed as type C. Meanwhile, all patients who did not undergo a further procedure due to an oversized defect (*n* = 6) were also diagnosed as type C. This result demonstrated that type C may reveal an oversized defect, indicating that a more rigorous assessment by our novel VSD classification strategy in intraoperative angiography is necessary. This method may eventually improve the success rate and avoid the damages caused by unnecessary procedure.

In conclusion, it is safe and effective to use this digital method to classify pmVSD with aneurysm as well as aiding in decision-making regarding interventions, thereby improving the effectiveness of interventional occlusion, and providing suggestions for transcatheter closure of pmVSD with aneurysm. Although the cases included in this study are limited, we will continue to expand this study in the future and explore more effective methods to improve the success rate and reduce complications. At the same time, regular follow-up for patients with postoperative arrhythmia is warranted.

## Figures and Tables

**Table 1:** Baseline data of various types of aneurysm.

	Types ($\bar{x}$ ± *s*)			*F*	*p*
	A (*n* = 54)	B (*n* = 51)	C (*n* = 123)		
*a* (mm)	8.25 ± 2.50	5.55 ± 3.07	6.66 ± 3.17	10.85	<0.01
*b* (mm)	6.49 ± 2.36	8.60 ± 3.43	7.74 ± 3.40	5.86	<0.01
*c* (mm)	4.29 ± 2.16	3.97 ± 2.90	10.46 ± 3.71	108.91	<0.01
*h* (mm)	6.45 ± 2.23	8.33 ± 2.46	7.92 ± 2.69	8.574	<0.01

*a*: the inlet portion of the aneurysm, *b*: the diameter of the midsegment of the aneurysm, *c*: the outlet portion of the aneurysm, and *h*: the height of the aneurysm.

## Data Availability

The data that support the findings of this study are available from the corresponding author, Zhiwei Zhang, upon reasonable request.
